# Laparoscopic-assisted myomectomy with uterine artery occlusion at a freestanding ambulatory surgery center: a case series

**DOI:** 10.1186/s10397-020-01075-2

**Published:** 2020-06-16

**Authors:** Paul MacKoul, Natalya Danilyants, Faraj Touchan, Louise Q. van der Does, Leah R. Haworth, Nilofar Kazi

**Affiliations:** The Center for Innovative GYN Care, 3206 Tower Oaks Blvd., Suite 200, Rockville, MD 20852 USA

**Keywords:** ASC, Ambulatory surgery center, BMI, Freestanding, laparoscopic-assisted myomectomy, Ligation, Minilaparotomy, Morbidly, Obese, occlusion

## Abstract

**Background:**

Non-hysteroscopic myomectomy is infrequently performed in a freestanding ambulatory setting, in part due to risks of intraoperative hemorrhage. There are also concerns about increased surgical risks for morbidly obese patients in this setting. The purpose of this study is to report the surgical outcomes of a series of laparoscopic-assisted myomectomy (LAM) cases at a freestanding ambulatory surgery center (ASC), including a comparative analysis of outcomes in morbidly obese patients (BMI > 40 kg/m^2^).

**Methods:**

A retrospective comparative analysis was performed of 969 women, age 18 years or older, non-pregnant, who underwent LAM by one of two high volume, laparoscopic gynecologic surgical specialists at a freestanding ambulatory surgery center serving the Washington, DC area, between October 2013 and February 2019. Reversible occlusion was performed laparoscopically by placing a latex-based rubber catheter as a tourniquet around the isthmus of the uterus, causing a temporary occlusion of the bilateral uterine arteries. Permanent occlusion was performed laparoscopically via retroperitoneal dissection and uterine artery ligation at the origin of the anterior branch of the internal iliac artery. Minilaparotomy was performed for specimen removal in all cases. No power morcellation was used. Postoperative complications were graded using the Clavien-Dindo Classification system. Outcomes were compared across BMI categories using Pearson Chi-Square.

**Results:**

Average myoma weight and size were 422.7 g and 8.3 cm, respectively. Average estimated blood loss (EBL) was 192.1 mL; intraoperative and grade 3 postoperative complication rates were 1.4% and 1.6%, respectively. While EBL was significantly higher in obese and morbidly obese patients, this difference was not clinically meaningful, with no significant difference in blood transfusion rates. There were no statistically significant intraoperative or postoperative complication rates across BMI categories. There was a low rate of hospital transfers (0.7%) for all patients.

**Conclusion:**

Laparoscopic-assisted myomectomy can be performed safely in a freestanding ambulatory surgery setting, including morbidly obese patients. This is especially important in the age of COVID-19, as elective surgeries have been postponed due to the 2020 pandemic, which may lead to a dramatic and permanent shift of outpatient surgery from the hospital to the ASC setting.

## Background

Uterine myomas are the most common gynecologic tumors in the USA, occurring in 20–50% of women of reproductive age [1, 2]. Symptomatic submucosal myomas are often treated by hysteroscopic resection, while leiomyomas with intramural or subserosal components are traditionally treated with myomectomy or hysterectomy [3, 4]. The three most common surgical approaches to myomectomy are conventional laparoscopic myomectomy (CLM), robotic-assisted laparoscopic myomectomy (RALM), and abdominal myomectomy (AM). Research shows CLM results in lower blood loss, fewer complications, shorter length of stay, and improved cosmesis compared to AM but longer operating times [5]. RALM parallels the advantages of CLM but with increased operative times and higher cost [6, 7].

Laparoscopic-assisted myomectomy (LAM) via minilaparotomy (3–5 cm incision) was first introduced in 1994 by Nezhat et al. as a way to overcome the inherent limitations of laparoscopy, while maintaining the advantages of a minimally invasive procedure [8]. The reported advantages of this hybrid approach include the ability to palpate the uterine body, remove larger myomas, and facilitate suturing of the uterine incision. Kalogiannidis et al. compared LAM to AM and found lower operative times, faster patient recovery, reduction of blood loss, shorter skin and uterine incisions, and decreased chance of postoperative adhesions [9]. Comparing LAM to laparoscopy, Prapas et al. also showed LAM had shorter operative times, as well as easier and faster uterine repair with a shorter incision and minimal requirement for electrocautery, an important factor in avoiding thermal tissue damage and maintaining the integrity of the uterus for future pregnancy [10]. The study also emphasized the ability to palpate the uterus during LAM in order to detect and excise smaller or deep intramural myomas that may be inadvertently left behind during laparoscopy, which may decrease the chance of recurrence. Indeed, studies comparing laparoscopy to abdominal myomectomy have shown higher recurrence rates of leiomyomas following laparoscopy, supporting the idea that the more complete the removal of myomas, the lower the chance of recurrence [11]. Our previous study comparing LAM to abdominal and laparoscopic myomectomy found similar results as the aforementioned studies, but also included a RALM group, which showed a greater rate of intraoperative complications, longer operative time, and smaller number and weight of myomas removed than LAM [12].

Hemorrhage remains a risk of any surgical myomectomy approach when removing these highly vascular tumors. Hickman et al. provided an evidence-based review of the numerous medical and surgical interventions that have been developed to minimize potentially significant blood loss during myomectomy [13]. These methods can be used in combination with any myomectomy surgical approach, and many of the techniques may be used together, providing an additive effect on decreasing intraoperative blood loss.

A meta-analysis by Tranoulis et al. focused on the myomectomy blood loss control technique of uterine artery occlusion (UAO), both permanent and transient [14]. The review included 12 studies comparing laparoscopic myomectomy with and without UAO and found a significant decrease in intraoperative blood loss, postoperative hemoglobin drop, and blood transfusion rate in the UAO groups. The UAO results also trended toward a shorter hospital stay and lower myoma recurrence rate than laparoscopy alone.

With rising rates of both obesity and ambulatory surgery in this country over the past 20 years, there has also been concern about increased surgical risks for obese patients in this setting. While there is evidence that patients with a body mass index (BMI) < 40 kg/m^2^ can safely undergo ambulatory surgery, and that there is increased risk of perioperative complications in the super obese (BMI > 50 kg/m^2^), there is limited data on the surgical outcomes for morbidly obese patients with BMI between 40 and 50 kg/m^2^ [15].

While past research has established the safety of same-day discharge following myomectomy, it is less frequently performed at a freestanding ambulatory surgery center (ASC), in large part due to the risk of hemorrhage [16]. The objective of the current study is to report the operative outcomes of a case series of 969 patients who underwent laparoscopic-assisted myomectomy at an ASC, using UAO for blood loss control. The patients were further grouped into three categories based on BMI: non-obese patients (BMI < 30 kg/m^2^), obese patients (BMI > 30 and < 40 kg/m^2^), and morbidly obese patients (BMI >40 kg/m^2^), and the comorbidities and surgical outcomes of each group were compared.

## Methods

A retrospective review was conducted of 969 consecutive patients who underwent LAM with UAO from October 2013 to February 2019 at an ASC serving the greater Washington, DC area. All cases were performed by one of two high-volume laparoscopic gynecologic surgical specialists from a single practice. All women in this case series were 18 years or older, non-pregnant, with symptomatic leiomyoma. IntegReview IRB, an independent institutional review board, deemed the review exempt from informed consent (IRB #CIGC-001). LAM was performed in all cases with transient uterine artery occlusion via pericervical tourniquet and/or permanent uterine artery ligation for blood loss control, as described below.

### LAM surgical technique

A 5-mm trocar is inserted at the umbilicus via direct entry followed by a 5-mm suprapubic port, with a third 5-mm LLQ trocar added in more complicated cases with very large leiomyomas, adhesions, or endometriosis. A ZUMI uterine manipulator (CooperSurgical, Inc., Trumbull, Connecticut) is used in all cases. Blood loss control measures consisted of transient uterine artery occlusion and/or permanent uterine artery occlusion via ligation at the origin of the anterior division of the internal iliac artery. A transient or permanent blood loss control method is determined based on the patient’s desire for fertility, uterine size, number of myomas, and complexity of the case.

### Transient uterine artery occlusion

Following laparoscopic entry, a latex rubber (or non-latex for patients with latex allergy) tourniquet is placed around the isthmus of the uterus, causing temporary occlusion of the bilateral uterine arteries. This limits blood flow and decreases the pulse pressure to the uterus.

Laparoscopic defects are made in the broad ligaments bilaterally, and the tourniquet is passed under direct visualization through the defects. The suprapubic incision is then extended either in a transverse or vertical direction (depending on the exposure needed) to 3–4 cm, followed by placement of a small or medium wound retractor. This incision allows the wound diameter to stretch to 6–8 cm, similar to a smaller open incision. The tourniquet is then tightened through the suprapubic incision.

### Permanent uterine artery occlusion

Following laparoscopic entry, the retroperitoneal spaces are opened, the uterine arteries are identified at the origin from the anterior division of the internal iliac artery, and uterine artery ligation is performed with a Harmonic scalpel (Ethicon, Inc., New Brunswick, New Jersey) which seals and divides the artery. The suprapubic incision is then extended, and a wound retractor is placed as described above.

Following uterine artery occlusion, leiomyomas are localized both visually and via palpation, and the uterine incision is made. Leiomyomas are then removed either intact or with manual segmentation above the fascia at the level of the skin, in order to minimize the risk of scattering leiomyomatous fragments into the abdominopelvic cavity. No electric morcellation is used.

The uterus can be externalized if needed, and uterine defects, including posterior, are hand-sewn and closed in layers using the standard abdominal myomectomy closure technique. In cases with transient uterine artery occlusion, the tourniquet is released upon closure of the uterine defect(s). Hemostasis is confirmed by laparoscopic survey at the end of the procedure. Fascia and skin incisions are closed using absorbable sutures.

Prior to surgery, postoperative instructions were reviewed in detail with the patient. After surgery, patients in the ASC were discharged home within 2–3 h of the end of their procedure. Pain management regimen included preoperative acetaminophen, intravenous narcotics intraoperatively and in the post-anesthesia care unit, and a prescription for oral narcotics and ibuprofen upon discharge.

Patients requiring transfer from the ASC to the hospital are transported via local medical transport company, with which the ASC has an agreement, at the expense of the ASC. The emergency department is contacted before transport to alert of the incoming patient, and the patient is transported directly to one of three local hospitals, all located within 10 miles of the ASC.

Postoperative phone calls are made by ASC staff on the day after discharge. If the patient cannot be reached, a follow-up phone call is made until the patient is reached or until postoperative day 7. All patients are scheduled for a 2-week postoperative visit.

The major indications for myomectomy included symptomatic leiomyomas causing pelvic pain, abnormal uterine bleeding with symptomatic anemia, pressure effect on bladder or bowel or pelvic veins, and infertility. Cases with concomitant procedures that are routinely performed during a myomectomy were included in the case series, such as adnexal surgery, adhesiolysis, cystoscopy, and resection of endometriosis. Cases with concomitant pelvic or abdominal surgery unrelated to the myomectomy, such as appendectomy, hernia repair, and pelvic organ prolapse procedures, were excluded from the case series because the added surgical time and increased risk of complications may skew the results. Presumed malignancies based on the pre-operative diagnosis were also excluded.

Data were retrieved from electronic medical records and included demographic and clinical patient characteristics, such as age, race, BMI, and previous abdominopelvic surgeries. The Elixhauser Comorbidity Index, a set of 30 comorbidity indicators used to predict hospital resource use and in-hospital mortality, was used to identify and record comorbid conditions that have been shown to potentially affect operative outcomes [17]. Operative outcomes included estimated blood loss (EBL); skin-to-skin operative time; number, size, type, and aggregate weight of leiomyomas; conversion from laparoscopy to standard laparotomy; intraoperative and postoperative complications, blood transfusions, and hospital transfers. Postoperative complications were graded using the Clavien-Dindo Classification system (Table 1) [18].

**Table 1:** Clavien-Dindo postoperative complication classification

Grade	Definition
**Grade I**	Any deviation from the normal postoperative course without the need for pharmacological treatment or surgical, endoscopic and radiological interventions Allowed therapeutic regimens are drugs as antiemetics, antipyretics, analgesics, diuretics, and electrolytes, and physiotherapy. This grade also includes wound infections opened at the bedside.
**Grade II**	Requiring pharmacological treatment with drugs other than such allowed for grade I complications. Blood transfusions and total parenteral nutrition are also included.
**Grade III**	Requiring surgical, endoscopic, or radiological intervention.
**IIIa**	Intervention not under general anesthesia.
**IIIb**	Intervention under general anesthesia.
**Grade IV**	Life-threatening complication (including CNS complications)^a^ requiring IC/ICU-management.
**IVa**	Single organ dysfunction (including dialysis).
**IVb**	Multi-organ dysfunction.
**Grade V**	Death of a patient.

*IC* intermediate care; *ICU* intensive care unit

^a^Brain hemorrhage, ischemic stroke, subarrachnoidal bleeding, but excluding transient ischemic attacks (TIA)

## Results

A total of 969 patients underwent LAM with UAO over the 5-year period. The number of patients with at least one prior abdominopelvic surgery was 39.7%, and 12.7% had at least one prior myomectomy (Table 2). Obesity rate was 32.4% (BMI > 30), of which 6.5% were morbidly obese with a BMI > 40. Two patients were considered super obese with a BMI of 52 and 74, respectively.

**Table 2:** Patient demographics

	***N*** = 969
**Age Group,*N* (%)**	
** < 30**	79 (8.2)
** 30–39**	626 (64.6)
** 40–49**	260 (26.8)
** 50–59**	3 (0.3)
**Race,*N*(%)**	
**White**	178 (18.4)
**Black**	491 (50.7)
**Other**	300 (30.9)
**Number of previous myomectomies,*N*(%)**	
**None**	366 (37.8)
**1**	106 (10.9)
**2 or more**	17 (1.8)
**Number of Previous Abdominal Surgeries,*N*(%)**	
**None**	574 (59.2)
**1**	263 (27.1)
**2**	81 (8.4)
**3 or more**	41 (4.2)
**Number of comorbidities,*N*(%)**	
**None**	353 (36.4)
**1**	334 (34.5)
**2**	176 (18.2)
**3 or more**	104 (10.7)
**BMI (kg/m**^**2**^**),*N*(%)**	
**< 30**	650 (67.1)
**30–39**	251 (25.9)

The surgeons performed UAO by transient tourniquet (TUAO) in 62.8% of cases; UAO by permanent uterine artery ligation (PUAO) in 12.3%. In 24.9% of the cases, both techniques were used in combination. Mean EBL was 192 mL (range 5–2000 mL) with 8 cases (0.7%) exceeding an EBL > 1000 mL (Table 3). The mean operative time was 70 min, with 80% of surgeries performed in under 90 min.

**Table 3:** Uterine artery occlusion method

	All (***N*** = 969)	Transient (***N*** = 609)	Permanent (***N*** = 119)	Both transient and permanent (***N*** = 241)
**BMI (kg/m2)—mean**	**27.9**	**27.3**	**29.5**	**28.5**
**Myoma weight (g)—mean**	**422.7**	**314.1**	**530.1**	**640.5**
**Estimated blood loss (ml)—mean**	**192.1**	**147.4**	**259.7**	**272.9**
**Intra-op complications, %**	**1.4**	**.3**	**3.4**	**3.3**
**Grade 1 post-op complications, %**	**2.7**	**2.0**	**4.2**	**3.7**
**Grade 2 post-op complications, %**	**3.6**	**1.5**	**6.7**	**7.5**
**Grade 3a post-op complications, %**	**0.6**	**.8**	**0.0**	**.4**
**Grade 3b post-op complications, %**	**1.0**	**.8**	**1.7**	**1.2**
**Blood transfusions, %**	**2.9**	**.7**	**5.9**	**7.1**

*UAO* uterine artery occlusion, *ml* milliliter, *min* minutes, *Intra-op* intraoperative, *Post-op* postoperative

Mean myoma weight removed was 422.7 g (range 0.1–3800 g). Mean number of myomas removed was 8.7 (range 1–100), and mean length of longest myoma was 8.3 cm (range 1–42 cm). The types of myomas removed were intramural (51.0%), submucosal (51.3%), subserosal (30.9%), and pedunculated (25.2%) (Table 4).

**Table 4:** Myoma characteristics

	N = 969
**Myoma Weight** (g), ***N*(%)**	
≤ 100	207 (21.4)
101–500	444 (45.8)
501–749	127 (13.1)
> 750	164 (16.9)
**Mean myoma weight** (g)	422.7
**Mean length** (cm)	8.3
**Subserosal** (%)	30.9
**Intramural** (%)	51.0
**Submucosal** (%)	51.3

*N* number, *g* grams, *cm* centimeters

As seen in Table 3, the overall rate of intraoperative complications was 1.4%, the most common of which were colon, bladder, or rectal defects, which were repaired during surgery without further complications. The rate of all postoperative complications (grades 1–3) was 7.9%. The rate of grade 3 postoperative complications (those requiring surgical, endoscopic, or radiological intervention) was 1.6%. No grade 4 or 5 postoperative complications occurred.

The most common postoperative complication was blood transfusion (grade 2), which occurred in 2.9% of patients. Three patients (0.3%) underwent hysterectomy in the immediate postoperative period for uncontrolled bleeding. Three patients required postoperative intrauterine balloon placement to control bleeding; two patients had abdominal hematoma that required surgical evacuation; one patient had a small bowel obstruction that was treated laparoscopically; and one patient required surgery for a pelvic abscess.

Seven patients (0.7%) required postoperative transfer to the hospital. Six were transferred for acute anemia requiring blood transfusion, one of which also required hysterectomy. One patient was transferred for an acute postoperative asthmatic reaction with low oxygen saturation. All patients recovered without further complication.

Comorbidities were compared across BMI categories: non-obese (BMI < 30), obese (BMI 30–39), and morbidly obese (BMI > 40) (Table 5). Not surprisingly, there was a significantly higher rate of hypertension, diabetes, and pulmonary comorbidities in the obese and morbidly obese groups, but the rate of cardiovascular disease (e.g., arrhythmia, clotting disorder, stroke, myocardial infarction) was not significantly different across BMI categories.

**Table 5:** Comorbidities per BMI category

Comorbidity category	Non-obese BMI < 30 (***N*** = 650)	Obese BMI 30–39 (***N*** = 251)	Morbidly obese BMI **>** 40 (***N*** = 63)	***P*** value**
**Cardiovascular**^**a**^**,*N*(%)**	40 (6.2)	13 (5.2)	4 (6.3)	.843
**Hypertension,*N*(%)**	22 (3.4)	43 (17.1)	14 (22.2)	.000
**Diabetes,*N*(%)**	3 (0.5)	7 (2.8)	2 (3.2)	.007
**Pulmonary**^**b**^**,*N*(%)**	80 (12.3)	42 (16.7)	17 (27.0)	.003

*BMI* body mass index (kg/m^2^)

**Pearson Chi-square

^**a**^Cardiovascular: arrhythmia, deep vein thrombosis, clotting disorder, coronary artery disease, myocardial infarction, stroke

^**b**^Pulmonary: chronic obstructive pulmonary disorder, asthma, sleep apnea, smoking

Surgical outcomes were also analyzed by BMI category (Table 6). There was no difference in operative times or rate of intraoperative or postoperative complications between BMI groups. The obese and morbidly obese groups had a higher mean EBL than non-obese patients (205 mL and 272 mL, respectively, compared to 180 mL, *P* = .000). However, these differences were not clinically meaningful, with no significant difference in blood transfusion rates (Table 7).

**Table 6:** Operative outcomes per BMI category

	Non-obese BMI < 30 (***N*** = 650)	Obese BMI 30–39 (***N*** = 251)	Morbidly obese BMI **>** 40 (***N*** = 63)	***P*** value*
**Mean BMI (kg/m**^**2**^**) (range)**	24.3 (14–29)	33.2 (30–39)	43.9 (40–70)	N/A
**Mean myoma weight (g) (range)**	407.9 (0–3800)	451.6 (0.5–3149)	477.7 (9–2800)	.418
**Mean operative time (min)**	68	70	72	.849
**Mean EBL (mL)**	179.8 (0–2000)	205.4 (10–2000)	272.3 (20–1500)	.000
**Blood transfusion, N (%)**	18 (2.8)	8 (3.2)	2 (3.2)	.564
**Intraoperative complications,*N*(%)**	10 (1.5)	4 (1.6)	0 (0.0)	.883
**Grade 1 postoperative complications,*N*(%)**	16 (2.5)	10 (4.0)	0 (0.0)	.178
**Grade 2 postoperative complications,*N*(%)**	20 (3.1)	11 (4.4)	4 (6.3)	.320
**Grade 3a postoperative complications,*N*(%)**	4 (0.6)	2 (0.8)	0 (0.0)	.772
**Grade 3b postoperative complications,*N*(%)**	7 (1.1)	2 (0.8)	1 (1.6)	0.845
**Transfer to hospital,*N*(%)**	5 (0.8)	2 (0.8)	0 (0.0)	.780

*N* number, *BMI* body mass index (kg/m^2^)

^a^Pearson Chi-square

**Table 7:** Most common postoperative complications per BMI category

	Non-obese BMI < 30 (***N*** = 650)	Obese BMI 30–39 (***N*** = 251)	Morbidly obese BMI **>** 40 (***N*** = 63)
**GRADE 1**			
Abdominal pain	7	3	0
Nausea/vomiting	3	2	0
Vaginal bleeding	2	0	0
Incisional bleeding	1	3	0
CO_2_ gas pain	1	0	0
Unable to void	1	1	0
Fever	1	0	0
Syncope	1	0	0
**GRADE 2**			
Blood transfusion	18	8	2
Incisional infection	2	3	2
UTI	2	0	0
Pulmonary embolism	1	0	0
**GRADE 3a**			
Incisional separation	2	2	0
Hemothorax	1	0	0
**GRADE 3b**			
Hematoma evacuation	2	0	0
Hysterectomy	2	1	0
Intrauterine balloon	1	1	1

## Discussion

Our study demonstrated that the laparoscopically-assisted approach to myomectomy, combined with transient and/or permanent uterine artery occlusion, allowed experienced surgeons to remove significant tumor loads while minimizing blood loss and complications even in morbidly obese patients. The low complication and transfusion rates reported in our case series are especially significant in this freestanding ambulatory setting, in which no sub-specialty back-up or blood transfusion capabilities exist.

Our LAM case series is unique in its use of uterine artery occlusion for blood control. In contrast, no medical or mechanical vasoconstriction was used in two prior LAM studies in which LAM was shown to have lower blood loss than abdominal myomectomy, but significantly more than the laparoscopic approach [9, 10]. Seidman et al. used vasopressin to control blood loss when comparing LAM to abdominal and laparoscopic myomectomy and found similar blood loss between the LAM and abdominal groups, but significantly more than the laparoscopic group [19]. Of note, the use of vasopressin has led to rare but serious consequences such as bradycardia, cardiovascular collapse, and even death [20–23]. All three of the above LAM studies showed LAM removed significantly more myomas than laparoscopic myomectomy and a similar amount of tumor removal to abdominal myomectomy.

In our case series, the minilaparotomy incorporated in the LAM approach allowed surgeons to not only remove large myomas, but also to excise a significant percentage of submucosal myomas. This is important, as submucosal myomas are associated with lower fertility rates, and studies have demonstrated that removing such myomas results in improvement in both conception and live birth rates [24]. While hysteroscopic resection is the classic treatment for women with submucosal myomas, patients with myomas in multiple locations require a different surgical approach. In our study, the LAM approach enabled the removal of as many submucosal (51.3%) as intramural myomas (51.0%). By comparison, a case series by Mallick et al. of 323 patients who underwent laparoscopic myomectomy showed the that majority of myomas removed were intramural (49%) and subserosal (33%), and less frequently submucosal (17%) and pedunculated (1%) [25].

The LAM approach also eliminates the need for electric morcellation, which is significant given the 2014 FDA safety warning against power morcellation due to the potential dissemination of undiagnosed malignancy [26]. Several survey and large state and national database review studies have examined the practice patterns of gynecologic surgeons since the FDA warning and found an increased rate of laparotomy and decrease in minimally invasive myomectomy [27–30]. However, a single-surgeon investigation by Pereira et al. in the 2 years before and after the FDA’s recommendation found no change in the ratio of abdominal to minimally invasive myomectomy [31]. Interestingly, however, the study did find a significant decrease in laparoscopic and robotic myomectomy, and a corresponding rise in LAM, which was performed without power morcellation.

The setting of our case series, a freestanding ambulatory surgery center, is also worthy of discussion. There has been a steady migration of minimally invasive gynecologic procedures to the ambulatory surgical setting, due to the reported advantages which include shorter perioperative and facility times, fewer delays, lower costs, lower risk of exposure to nosocomial infections, intensified quality control processes, customized surgical environments, and highly specialized surgical staff [32–36].

Also, while previous studies of ASCs have emphasized the importance of appropriate patient selection [37], such strict pre-selection did not occur at our ASC. There is a prevailing yet unproven caution against performing ambulatory surgery on patients with morbid obesity due to the associated comorbidities such as obstructive sleep apnea, and the potential for increased risk of perioperative complications [15]. In our morbidly obese patients, which accounted for 6.5% of our case series, there was a higher incidence of hypertension, diabetes, and pulmonary comorbidities, but the rate of cardiovascular comorbidities was not significantly different than the non-obese group. Morbidly obese patients were able to successfully undergo surgery in this case series, with no difference in operative time, intra- and postoperative complications, blood transfusions, or hospital transfers.

The transition of outpatient surgery from the hospital to the ASC setting is more important now than ever in light of the COVID-19 pandemic. With many patients having much-needed, but not emergent surgeries postponed due to the pandemic, ASCs are in a prime position to take on this backlog of elective procedures. With the low overhead and high level of patient care quality, and the fact that ASCs have been largely isolated from COVID-19 responses, these facilities may soon become the primary setting for elective procedures. A shift in surgical setting will also help to reduce pressure on hospitals and limit patient exposure to COVID-19 treatment environments. The 2020 pandemic will likely lead to an acceleration of the migration of outpatient surgery from the hospital to ASCs, potentially changing the landscape of the ambulatory surgery setting forever.

### Strengths and limitations

The strength of the current study is the large number of consecutive cases, 6.5% of which included morbidly obese patients. To our knowledge, this is also the largest case series on LAM, as well as the largest case series at a freestanding ASC. However, the retrospective nature of this case series is limited by the availability and accuracy of the medical records. All postoperative complications reported to these surgeons were captured, but patients who may have sought treatment at a different facility or physician’s office may not be reflected in our postoperative complication data. It is also important to recognize the significance of surgeon skill and experience in relation to surgical outcomes. Studies have shown an association between high-volume surgeons and lower complication rates, as well as higher rates of same-day discharge [38, 39]. The surgeons in this study are experienced, gynecologic laparoscopic specialists, who each perform more than 100 myomectomies per year, and whose outcomes, therefore, may not be representative of the general gynecologic surgical community.

## Conclusion

Our case series showed that LAM, in combination with the blood loss control technique of uterine artery occlusion, enabled experienced surgeons to remove a large amount of myomas while effectively preventing hemorrhage and minimizing complications. This control over blood loss is especially important in a freestanding ambulatory surgery center, where intraoperative blood transfusion capability does not exist. Our series also demonstrated the safety of performing surgery on morbidly obese patients in an ASC. As the demand for outpatient surgery grows, especially in the age of COVID-19, so will the importance of evaluating the safety of complicated surgeries and high-risk patients in the freestanding ambulatory setting.

## Data Availability

All data has been summarized in the tables in the manuscript. Raw data is available upon request from the corresponding author.

## Abbreviations

CLM: Conventional laparoscopic myomectomy
RALM: Robotic-assisted laparoscopic myomectomy
AM: Abdominal myomectomy
LAM: Laparoscopic-assisted myomectomy
UAO: Uterine artery occlusion
TUAO: Transient uterine artery occlusion
PUAO: Permanent uterine artery occlusion
BMI: Body mass index
ASC: Freestanding ambulatory surgery center
EBL: Estimated blood loss
